# Supplementary material for: Elevated IL-6 and IL-22 in Early Pregnancy Are Associated with Worse Disease Course in Women with Inflammatory Bowel Disease
Source: Int J Mol Sci. 2022 Sep 7;23(18):10281. doi: 10.3390/ijms231810281 (PMC9499187; doi:10.3390/ijms231810281)
Supplement: Supplementary file 1 [file ijms-23-10281-s001.zip › ijms-1852059-supplementary.pdf]

Supplemental Table S1: cytokine changes associated with post-partum active CD flare-up

Cytokines	t-statistic	*P*-value
CRP	0.0121	0.991
IFN-γ	-1.6643	0.117
IL-27	0.1539	0.880
IL-10	1.2652	0.225
IL-12p70	0.3870	0.706
IL-13	-1.0466	0.318
IL-1β	0.0710	0.944
IL-2	0.4056	0.694
IL-21	0.8751	0.396
IL-22	-0.8174	0.427
IL-23	-0.7227	0.484
IL-4	-1.0392	0.323
IL-6	-1.2810	0.220
IL-8	-0.7335	0.475
TNFα	-0.2740	0.788
IL-5	1.0990	0.300
IL-1α	-0.4284	0.680
IL-17	0.9483	0.360
IL-12p40	0.2637	0.796

Supplemental Table S2: cytokine changes associated with post-partum active UC flare-up

Cytokines	t-statistic	*P*-value
CRP	-1.2534	0.229
IFN-γ	-1.1054	0.286
IL-27	-0.9232	0.371
IL-10	0.1881	0.853
IL-12p70	0.8903	0.391
IL-13	0.0352	0.972
IL-1β	-1.0558	0.308
IL-2	0.7084	0.493
IL-21	-1.3348	0.202
IL-22	-0.9264	0.370
IL-23	-0.1627	0.873
IL-4	1.2186	0.242
IL-6	-1.0082	0.329
IL-8	-1.5161	0.150
TNFα	-1.3483	0.198
IL-5	-2.1517	0.121
IL-1α	-1.2980	0.251
IL-17	-1.1324	0.278
IL-12p40	-1.7294	0.104

Supplemental Table S3: correlation between T1 cytokines and pregnancy outcomes among women with UC

T1 cytokines	Preterm	C-sections	Planned C-sections
CRP	-0.065	0.028	-0.306
IFN-γ	0.389	-0.585*	-0.131
IL-27	0.065	-0.362	-0.306
IL-10	-0.194	-0.53	-0.48
IL-12p70	-0.174	-0.646*	-0.174
IL-13	-0.298	-0.581	-0.4
IL-1β	-0.224	-0.075	-0.2
IL-21	0	-0.585*	-0.218
IL-22	-0.149	0.129	0.1
IL-23	-0.13	-0.53	-0.131
IL-4	-0.259	-0.251	-0.393
IL-6	-0.324	-0.474	-0.48
IL-8	0.13	-0.418	0.131
TNF-α	-0.194	-0.753**	-0.48
IL-17	-0.453	-0.585*	-0.131
IL-12p40	-0.324	-0.362	0.306

Pearson correlation coefficients, $^*p < 0.05$, $^{**}\ p < 0.01$.

Supplemental Table S4: correlation between T1 cytokines and pregnancy outcomes among women with CD

T1 cytokines	Birth weight	C-section
TNFα	-0.221	-0.147
IFN-γ	-0.256	0.098
IL-1α	-0.234	0.507
IL-1β	-0.2	0.44
IL-12p40	0.102	0.489
IL-12p70	-0.286	-0.075
IL-2	0.343	0.275
IL-4	0.018	0
IL-5	0.638	0.291
IL-10	-0.102	-0.342
IL-13	-0.333	0
IL-17	-0.095	0.195
IL-21	0.06	0.049
IL-22	-0.151	0.519
IL-23	0.155	-0.075
IL-27	-0.508	0.098
CRP	-0.214	-0.049
IL-6	-0.256	0.244
IL-8	-0.34	0.733**

Pearson correlation coefficients, $^{**}\ p < 0.01$.
